# Lauren classification-based combined model integrating clinicopathological and spectral CT features for disease-free survival prediction in gastric cancer

**DOI:** 10.1186/s40644-026-01064-2

**Published:** 2026-06-03

**Authors:** Haibo You, Tiezhu Ren, Min Xu, Qianqian Chen, Long Ma, Yue Peng, Chenyang Zhang, Xinyu Liu, Wenjuan Zhang

**Affiliations:** 1 Department of Radiology, The Second Hospital & Clinical Medical School, Lanzhou University, Cuiyingmen No. 82, Chengguan District, Lanzhou, 730030 China; 2 Gansu Medical Imaging Science Data Center, Lanzhou, 730030 China; 3 Gansu International Scientific and Technological Cooperation Base of Medical Imaging Artificial Intelligence, Lanzhou, China

**Keywords:** Gastric cancer, Lauren classification, Spectral CT, Disease-free survival, Prediction model

## Abstract

**Background:**

The Lauren classification (intestinal versus diffuse type) is pivotal for prognosing gastric cancer and guiding treatment selection. This study developed an integrated preoperative prediction model that merges preoperatively available clinicopathological characteristics with spectral CT parameters to distinguish Lauren subtypes and predict disease-free survival (DFS).

**Methods:**

This single-centre, retrospective diagnostic-accuracy study included 224 chemotherapy-treated gastric cancer patients with histopathologically confirmed intestinal or diffuse-type Lauren classification and two-year disease-free survival outcomes. Portal-venous phase spectral computed tomography (CT) parameters (CT₇₀ₖₑ_v_, iodine concentration, and normalized iodine concentration) and clinicopathological variables (tumor differentiation and Borrmann type) were assessed. Independent predictors were identified using multivariate logistic regression, and three models were constructed: clinicopathological, spectral CT, and combined. Diagnostic performance was evaluated using receiver operating characteristic (ROC) analysis, calibration curves, decision curve analysis, and 1000-bootstrap internal validation. Disease-free survival was further analysed using Kaplan–Meier curves, the log-rank test, and Cox proportional hazards regression.

**Results:**

Multivariate analysis identified poor differentiation (OR = 9.80, P < < 0.001) and Borrmann type III/IV (OR = 2.65, *P* = 0.004) as clinicopathological predictors, while portal-venous phase CT at 70 keV (OR = 1.34, P < < 0.001), iodine concentration (OR = 2.08, *P* = 0.001), and normalized iodine concentration (OR = 2.09, P < < 0.001) were significant spectral CT predictors of diffuse-type histology. The combined model demonstrated an area under the curve (AUC) of 0.901 (95% CI: 0.859–0.944) for Lauren classification, outperforming both the pathological model (AUC = 0.810) and the spectral CT model (AUC = 0.861). The optimism-corrected AUC was 0.901 (95% CI: 0.857–0.943) based on 1000-bootstrap validation. For disease-free survival (DFS) prediction, the model achieved an AUC of 0.896 (95% CI: 0.851–0.941) at a cut-off value of 0.646, with an optimism-corrected AUC of 0.895 (95% CI: 0.850–0.939). Calibration curves and decision curve analysis indicated satisfactory agreement and a superior net benefit. Kaplan-Meier analysis revealed significant separation between high-risk and low-risk groups (2-year DFS rates: 7.2% versus 84.4%; log-rank chi2 = 158.88, P < < 0.001), and Cox regression analysis confirmed a substantially increased recurrence risk in the high-risk group (HR = 10.97, 95% CI: 6.68–17.99, P < < 0.001).

**Conclusion:**

The integrated model provides accurate preoperative Lauren classification and robust DFS prediction, serving as a multimodal tool for gastric cancer risk stratification and treatment planning.

## Introduction

Gastric cancer is the fifth most common malignancy and the fifth leading cause of cancer-related mortality worldwide [1]. In China, it represents the third leading cause of cancer death and accounts for nearly half of global cases [2, 3]. Most patients are diagnosed at an advanced stage [2]. Lauren’s classification, established in 1965, remains a fundamental histopathological system that stratifies gastric cancer into intestinal and diffuse types [4]. These subtypes exhibit distinct biological behaviors [4, 5]. The intestinal type is characterized by glandular formation, cohesive growth patterns, and a relatively favorable prognosis [4, 6]. In contrast, the diffuse type is characterized by loss of cell adhesion, signet-ring cells, and aggressive metastatic potential, leading to poor outcomes [4–6].

Substantial evidence demonstrates that Lauren’s classification significantly influences therapeutic decision-making [4, 6, 7]. Intestinal-type tumors generally respond more favorably to fluoropyrimidine–platinum chemotherapy and trastuzumab [5, 7], whereas diffuse-type tumors often exhibit primary resistance to conventional cytotoxic agents and are associated with earlier peritoneal dissemination [6, 8, 9]. Consequently, accurate preoperative identification of the Lauren subtype is critical for prognosis assessment and treatment planning [7, 9].

The current Lauren classification relies exclusively on histopathological examination of endoscopic biopsies or surgical specimens [7, 8]. This method is limited by sampling heterogeneity, as biopsy specimens may not accurately represent the entire tumor due to spatial variation within the lesion [7, 9]. Comparative studies have reported significant discordances in the Lauren classification between biopsy samples and matched surgical specimens, which may compromise therapeutic strategies [7–9]. Additionally, the inability to assess Lauren subtype non-invasively restricts dynamic monitoring during neoadjuvant therapy or follow-up [8, 9]. Identification of a non-invasive biomarker for Lauren classification could enable preoperative risk stratification and personalized treatment selection without the need for invasive procedures [7, 9].

Spectral computed tomography (CT) offers significant potential as a non-invasive biomarker by simultaneously acquiring low- and high-energy data, enabling quantitative iodine mapping that reflects tumor vascularity and microenvironmental heterogeneity [10, 11]. Parameters such as iodine concentration (IC), normalized iodine concentration (NIC), and effective atomic number have demonstrated correlations with histopathological features in gastric cancer [10, 12]. Intestinal-type tumors typically exhibit more extensive vascular networks and greater iodine uptake, whereas diffuse-type tumors display desmoplastic stromal reactions with altered perfusion characteristics [11, 12]. Portal-venous phase imaging captures the distribution of contrast agent within the tumor microvasculature and interstitial space, potentially corresponding to the distinct architectural patterns of intestinal and diffuse subtypes [10, 12, 13]. Despite these associations, the relationship between portal-venous phase spectral CT metrics and Lauren classification remains largely unexplored [10, 13]. Advances in CT texture analysis have further enhanced spectral CT’s capabilities [13, 14]. Texture analysis has demonstrated significant associations with key biomarkers, including differentiation grade and Lauren type, providing a biological basis for imaging–molecular correlations [14, 15]. Recent studies indicate that dual-energy CT-based radiomics signatures can predict Lauren classification preoperatively, suggesting a transferable method for stratifying gastric cancer [10, 13]. Additionally, spectral CT parameters have shown promise in predicting disease-free survival (DFS) by reflecting tumor aggressiveness and micrometastatic potential [12, 15].

The Borrmann classification is an established endoscopic and radiological system that characterizes tumor growth patterns and biological behavior [16]. Recent studies have identified strong associations between the Borrmann classification and the Lauren subtype [16, 18]. Borrmann types I and II (polypoid and fungating) predominantly correspond to intestinal-type gastric cancer with expansive growth, whereas types III and IV (ulcerative infiltrative and diffusely infiltrative/linitis plastica) are closely associated with diffuse-type histology, infiltrative growth, and desmoplasia [16–18]. Notably, Borrmann type IV strongly predicts diffuse-type gastric cancer with features of epithelial-mesenchymal transition [17, 18]. These associations suggest that Borrmann type could serve as a clinically accessible surrogate for Lauren classification [16].

These insights inform the objective of our study. We aim to develop and validate a cost-effective, integrated preoperative prediction model that combines routinely available clinicopathological characteristics, including Borrmann type and tumor differentiation, with portal-venous phase spectral CT parameters [10, 13, 16]. The primary goal is to identify Lauren subtypes and predict disease-free survival in gastric cancer [12, 15, 17]. We hypothesize that a combined model utilizing these multimodal parameters will outperform any single conventional or imaging parameter [10, 13]. This model could provide a robust tool for preoperative risk stratification and prognosis, thereby guiding treatment selection and follow-up strategies [4, 6, 16].

## Materials and methods

### Study design and ethical approval

This single-centre, retrospective diagnostic-accuracy study was approved by the Ethics Committee of Lanzhou University Second Hospital (approval number: 2025 A-1258) with a waiver of informed consent due to its retrospective nature [19]. The study complied with the Declaration of Helsinki [20]. Clinical trial number: not applicable. Reporting followed the STARD 2015 guidelines [21] and the TRIPOD statement for diagnostic prediction models [22].

### Patient selection

A total of 224 consecutive gastric cancer patients treated with chemotherapy, diagnosed between January 2021 and January 2024, were enrolled. Inclusion criteria were: (1) adults (≥ 18 years); (2) biopsy-confirmed gastric cancer with histopathologically confirmed intestinal or diffuse-type Lauren classification based on surgical specimens; (3) known two-year survival outcomes; (4) baseline spectral CT imaging available; and (5) all patients received chemotherapy treatment. Exclusion criteria included patients who did not receive chemotherapy, those who received non-chemotherapy antitumor therapies (e.g., targeted therapy, immunotherapy) as initial treatment, patients with inadequate image quality, tumours too small for segmentation, missing data, or mixed-type/indeterminate Lauren classification.

Disease-free survival definition. Disease-free survival (DFS) was defined as the time interval from the date of surgical resection to the date of first documented recurrence or death from any cause, whichever occurred first. Recurrence events included local recurrence (anastomotic or regional lymph node recurrence), distant metastasis (hepatic, pulmonary, bone, or other extra-abdominal metastases), and peritoneal dissemination (ascites with positive cytology or peritoneal nodules confirmed by imaging).

Follow-up protocol. All patients underwent standardized postoperative surveillance according to institutional protocol. Follow-up evaluations were scheduled at 6-month intervals for the first 24 months after surgery, with each visit including clinical assessment, laboratory tests, and contrast-enhanced CT of the chest, abdomen, and pelvis. Additional imaging (MRI or PET-CT) and/or histopathological biopsy were performed when recurrence was suspected. The date of recurrence was recorded as the time of the scheduled follow-up visit at which recurrence was first documented. Patients who remained recurrence-free at the 24-month visit were censored at that time point.

### CT protocol

Imaging was performed using a 256-detector Revolution CT (GE Healthcare) in gemstone spectral-imaging mode. After a six-hour fast and ingestion of 500–1000 ml of water, supine scans were obtained from the diaphragm to the lower poles of the kidneys. Acquisition parameters were as follows: 375 mAs, 80–140 kVp fast switching, 0.6 s rotation, pitch 0.992, 512 × 512 matrix, and 1.25 mm slice thickness. Iohexol (320 mg I/ml) was administered at a rate of 3–4 ml/s (1.0 ml/kg). Both arterial (25–30 s) and portal-venous (50–60 s) phases were acquired.

### Image analysis

Two abdominal radiologists, with 5 and 7 years of experience respectively, independently delineated circular regions of interest (ROIs) on the most representative tumour section. Necrotic areas, vessels, and folds were excluded. The mean values from both readers were used for subsequent analysis. Spectral parameters assessed in both phases included: CT attenuation at 40 keV and 70 keV, iodine concentration (IC), normalised iodine concentration (NIC = IClesion / ICaorta), effective atomic number (Eff-z), and normalized effective atomic number(NEff-z = Eff-zlesion / Eff-zaorta).

### Statistical analysis

Statistical analyses were performed using SPSS 30.0 (IBM) and R 4.5.2. Normality was assessed with the Shapiro–Wilk test. Parametric data are presented as mean ± standard deviation and compared using the independent t-test. Nonparametric data are reported as medians (interquartile ranges) and compared using the Mann–Whitney U test. Categorical variables were compared using the chi-squared test or Fisher’s exact test. Inter-reader agreement was quantified using intraclass correlation coefficients (ICC).

Univariate logistic regression was performed to identify potential predictors of diffuse-type histology and disease-free survival. Variables with *P* < 0.05 in univariate analysis were subsequently entered into multivariate logistic regression using the enter method to identify independent predictors. For spectral CT parameters, collinearity was assessed using variance inflation factor (VIF); parameters with VIF > 10 were excluded from the final multivariate model to ensure stability.

Three prediction models were constructed: (1) a clinicopathological model incorporating independent predictors identified from multivariate analysis of clinicopathological characteristics (poor differentiation and Borrmann type III/IV); (2) a spectral CT model incorporating independent spectral CT parameters (portal-venous phase CT₇₀ₖₑ_v_, iodine concentration [IC], and normalized iodine concentration [NIC]); and (3) a combined model integrating all independent predictors retained in the respective multivariate analyses.

Diagnostic performance for Lauren classification and disease-free survival (DFS) prediction was evaluated with receiver operating characteristic (ROC) curves, reporting area under the curve (AUC), sensitivity, specificity, positive predictive value (PPV), negative predictive value (NPV), and 95% confidence intervals. Optimal thresholds were identified using the Youden index. Pairwise AUC comparisons between each individual model and the combined model were performed using DeLong’s method in R 4.5.2. Model calibration was assessed using calibration curves and Brier scores for both Lauren classification and DFS prediction. Decision curve analysis (DCA) was performed to evaluate the net clinical benefit of each prediction model across different threshold probabilities for both endpoints. A two-tailed P value < 0.05 was considered statistically significant.

Internal validation. To assess model stability and quantify overfitting, internal validation was performed using 1000-bootstrap resampling. In each iteration, 224 patients were randomly sampled with replacement from the original cohort, and the area under the curve (AUC) was recalculated. The optimism-corrected AUC was estimated by subtracting the mean optimism (difference between bootstrap apparent performance and test performance) from the original AUC. Bootstrap 95% confidence intervals were derived from the 2.5th and 97.5th percentiles of the resampled AUC distribution.

Survival analysis. Disease-free survival was analyzed using the Kaplan-Meier method, and differences between risk groups were compared using the log-rank test. Univariate Cox proportional hazards regression was performed to evaluate the association between the combined model risk group and DFS. The proportional hazards assumption was assessed by visual inspection of log-minus-log survival plots.

## Results

### Patient characteristics and clinicopathological predictors

A total of 224 patients with gastric cancer were included, with 89 (39.7%) classified as intestinal-type and 135 (60.3%) as diffuse-type. Significant differences between groups were identified in sex distribution (*P* = 0.036), serum CEA levels (*P* = 0.048), tumor differentiation (*P* < 0.001), and Borrmann type (*P* < 0.001) (Table 1). Multivariate logistic regression indicated that poor differentiation (OR = 9.80, 95% CI: 5.15–18.65, *P* < 0.001) and Borrmann type III/IV (OR = 2.65, 95% CI: 1.37–5.09, *P* = 0.004) were associated with diffuse-type histology (Table 2).

**Table 1:** Baseline clinicopathological characteristics of the study cohort

Characteristics		Intestinal type (*n* = 89)	Diffuse type (*n* = 135)	t/z/x²	*P*- value
Age (y, M ± SD)		58.56 ± 7.91	57.85 ± 9.18	0.598	0.551
Sex (n, %)				4.394	0.036^*^
	Male	80(89.89%)	107(79.26%)		
	Female	9(10.11%)	28(20.74%)		
CA199[median (IQR)]		10.70(6.09,21.20)	8.71(5.28,14.70)	-1.232	0.218
CA125[median (IQR)]		9.37(6.29,13.40)	10.40(7.06,16.00)	1.827	0.068
CEA[median (IQR)]		2.28(1.50,5.43)	1.96(1.27,3.57)	-1.975	0.048^*^
AFP[median (IQR)]		2.45(1.92,3.25)	2.31(1.61,3.19)	-1.370	0.171
Differentiation(%)				61.436	<0.001^*^
	Non-poor	63(70.79%)	25(18.52%)		
	poor	26(29.21%)	110(81.48%)		
Tumor location (%)				4.049	0.132
	cardia	28(31.46%)	27(20.00%)		
	corpus	29(32.58%)	47(34.81%)		
	pyloric antrum	32(35.96%)	61(45.19%)		
Borrmann type (%)				16.704	<0.001^*^
	Ⅰ or Ⅱ	47(52.81%)	35(25.93%)		
	Ⅲ or IV	42(47.19%)	100(74.07%)		

Legend: CPS refers to Combined Positive Score; CEA denotes carcinoembryonic antigen; CA125 and CA199 indicate carbohydrate antigens 125 and 199, respectively; AFP represents alpha-fetoprotein

**Table 2:** Univariate and multivariate logistic regression analysis of clinico-pathological characteristics

Characteristics		Univariate analysis		Multivariate analysis	
		OR (95% CI)	*P*-value	OR (95% CI)	*P*-value
Age		0.99(0.96, 1.02)	0.549		
Sex		0.65(0.28, 1.50)	0.311		
CA199		1.00(1.00, 1.00)	0.926		
CA125		1.02(1.00, 1.06)	0.102		
CEA		1.00(1.00, 1.00)	0.968		
AFP		1.00(1.00, 1.00)	0.248		
Differentiation					
	Non-poor	Ref.			
	Poor	10.66(5.68, 20.03)	<0.001^*^	9.80(5.15, 18.65)	<0.001^*^
Tumor location					
	cardia	Ref.			
	corpus	1.68(0.83, 3.39)	0.148		
	pyloric antrum	1.98(1.00, 3.90)	0.050^*^		
Borrmann type					
	Ⅰ or Ⅱ	Ref.			
	Ⅲ or IV	3.20(1.81, 5.64)	<0.001^*^	2.65(1.37, 5.09)	0.004^*^

Legend: Only significant variables in univariate analysis (^*^ represents P<0.05) were included in multivariate analysis. OR = Odds ratio; CI = Confidence interval; Ref. = Reference category. CEA denotes carcinoembryonic antigen; CA125 and CA199 indicate carbohydrate antigens 125 and 199, respectively; AFP represents alpha-fetoprotein

### Spectral CT parameters and reproducibility

Significant differences between groups were observed in portal-venous phase parameters, including CT₄₀ₖₑ_v_ (*P* = 0.015), CT₇₀ₖₑ_v_ (P < < 0.001), iodine concentration (*P* = 0.007), normalized iodine concentration (P < < 0.001), and effective atomic number (*P* = 0.034). No significant differences were found in arterial-phase parameters (Table 3).

**Table 3:** Comparison of spectral CT parameters of the study cohort

	Parameter	Intestinal type (*n* = 89)	Diffuse type (*n* = 135)	t/z	*P*-value
Arterial-phase					
	CT_40kev_	151.52(125.89,187.93)	150.95(125.40,193.50)	-0.189	0.850
	CT_70kev_	68.08(56.46,80.87)	68.57(58.26,85.60)	0.669	0.504
	IC	16.10(11.08,19.32)	15.89(10.70,19.06)	-0.708	0.479
	NIC	0.12(0.09,0.15)	0.11(0.08,0.15)	-1.099	0.272
	Eff-z	8.58(8.38,8.72)	8.59(8.34,8.71)	-0.214	0.831
	NEff-z	0.70(0.67,0.73)	0.70(0.67,0.74)	0.369	0.712
Portal-venous phase					
	CT_40kev_	176.71(154.05,201.23)	184.79(164.40,222.60)	2.434	0.015^*^
	CT_70kev_	72.12 ± 17.08	88.47 ± 20.02	-6.333	<0.001^*^
	IC	17.70(16.16,20.36)	18.75(16.91,23.48)	2.704	0.007^*^
	NIC	0.39(0.32,0.45)	0.53(0.44,0.63)	7.036	<0.001^*^
	Eff-z	8.68(8.57,8.77)	8.71(8.60,8.93)	2.121	0.034^*^
	NEff-z	0.89(0.87,0.91)	0.89(0.87,0.91)	0.292	0.770

Legend: The parameters include CT values at 40 and 70 keV (HU), X-ray photon energy shift (λHu), iodine concentration (IC), normalized iodine density (NIC), and effective atomic number (Eff-z). CPS refers to Combined Positive Score, HU denotes Hounsfield Units, IC represents iodine concentration, and NIC indicates normalized iodine density (^*^ represents P<0.05)

Univariate analysis demonstrated associations between PVP-CT₄₀ₖₑ_v_, PVP-CT₇₀ₖₑ_v_, PVP-IC, and PVP-NIC and Lauren classification (Table 4). Multivariate analysis identified PVP-CT₇₀ₖₑ_v_ (OR = 1.34, 95% CI: 1.23–1.45, P < < 0.001), PVP-IC (OR = 2.08, 95% CI: 1.33–3.27, *P* = 0.001), and PVP-NIC (OR = 2.09, 95% CI: 1.41–3.11, P < < 0.001) as independent predictors (Table 4).

**Table 4:** Univariate and multivariate logistic regression analysis of Spectral CT parameters

		Univariate analysis		Multivariate analysis	
	Parameter	OR (95% CI)	*P*-value	OR (95% CI)	*P*-value
Arterial-phase					
	CT_40kev_	1.00(1.00,1.01)	0.838		
	CT_70kev_	1.01(0.99,1.02)	0.423		
	IC	0.99(0.95,1.04)	0.800		
	NIC	1.16(0.79,1.69)	0.452		
	Eff-z	0.96(0.38,2.39)	0.926		
	NEff-z	1.21(0.73,2.01)	0.456		
Portal-venous phase					
	CT_40kev_	1.01(1.00,1.01)	0.018^*^	Excluded < a	
	CT_70kev_	1.05(1.03,1.07)	<0.001^*^	1.34(1.23,1.45)	<0.001^*^
	IC	1.08(1.02,1.14)	0.008^*^	2.08(1.33,3.27)	0.001^*^
	NIC	2.01(1.58,2.57)	<0.001^*^	2.09(1.41,3.11)	<0.001^*^
	Eff-z	3.02(1.02,8.95)	0.046^*^	Not retained < b	
	NEff-z	0.86(0.40,1.83)	0.696		

Legend: Only significant variables in univariate analysis (^*^ represents *P* < 0.05) were included in multivariate analysis. OR = Odds ratio; The parameters include CT values at 40 and 70 keV (HU), iodine concentration (IC), normalized iodine density (NIC), and effective atomic number (Eff-z).HU denotes Hounsfield Units, IC represents iodine concentration, and NIC indicates normalized iodine density (^*^ represents *P* < 0.05). a Excluded from the final model due to severe multicollinearity (VIF = 12.369).b Not retained in the final multivariate model (*P* = 0.110)

Intraclass correlation coefficients for spectral CT parameters ranged from 0.871 to 0.935 (Tables 5 and 6). Collinearity diagnostics revealed severe multicollinearity for CT₄₀ₖₑ_v_ (VIF = 12.369), which was consequently excluded from the final model. All parameters retained in the final multivariate model—namely CT₇₀ₖₑ_v_ (VIF = 3.986), IC (VIF = 4.084), and NIC (VIF = 1.928)—demonstrated VIF values well below the conventional threshold of 5 (Table 7).

**Table 5:** Inter-observer reproducibility of spectral CT arterial-phase parameters

Parameter	Arterial-phase		
	Observer 1	Observer 2	ICC
CT_40kev_	151.03(124.90,191.34)	149.03(127.45,193.94)	0.921(0.899,0.939)
CT_70kev_	67.54(56.96,82.74)	68.13(58.10,83.24)	0.917(0.894,0.936)
IC	14.85(11.05,19.72)	14.59(11.04,19.08)	0.935(0.916,0.949)
Eff-z	8.57(8.36,8.74)	8.59(8.36,8.73)	0.888(0.856,0.913)

**Table 6:** Inter-observer reproducibility of spectral CT portal-venous phase parameters

	Portal-venous phase		
Parameter	Observer 1	Observer 2	ICC
CT_40kev_	180.96(158.14,215.47)	182.79(158.07,219.82)	0.896(0.866,0.919)
CT_70kev_	80.01(67.32,90.87)	80.71(68.57,92.61)	0.909(0.884,0.930)
IC	18.17(16.17,22.61)	18.36(16.19,23.11)	0.871(0.835,0.899)
Eff-z	8.69(8.56,8.89)	8.70(8.59,8.90)	0.879(0.845,0.905)

Legend: AP = arterial phase; VP = portal venous phase; CT_40kev_ / CT_70kev_ = virtual monoenergetic CT values at 40 keV and 70 keV; IC = iodine concentration; Eff-z = effective atomic number. ICC values close to 1 reflect excellent agreement

**Table 7:** Collinearity diagnostic of spectral CT modeling variables

Parameter	Tolerance	Variance inflation factor	Included in final model
CT_40keV_VP_	0.081	12.369	No
CT_70kev−VP_	0.251	3.986	Yes
IC_VP_	0.245	4.084	Yes
NIC_VP_	0.519	1.928	Yes

Legend: Collinearity diagnosis was performed on variables retained in the final multivariate model after excluding CT₄₀ₖₑᵥ (VIF > 10). VIF > 10 indicates strong collinearity; 3 ≤ VIF ≤ 10 indicates slight collinearity; VIF < 3 indicates no collinearity

### Diagnostic performance for Lauren classification

**Fig. 1:**
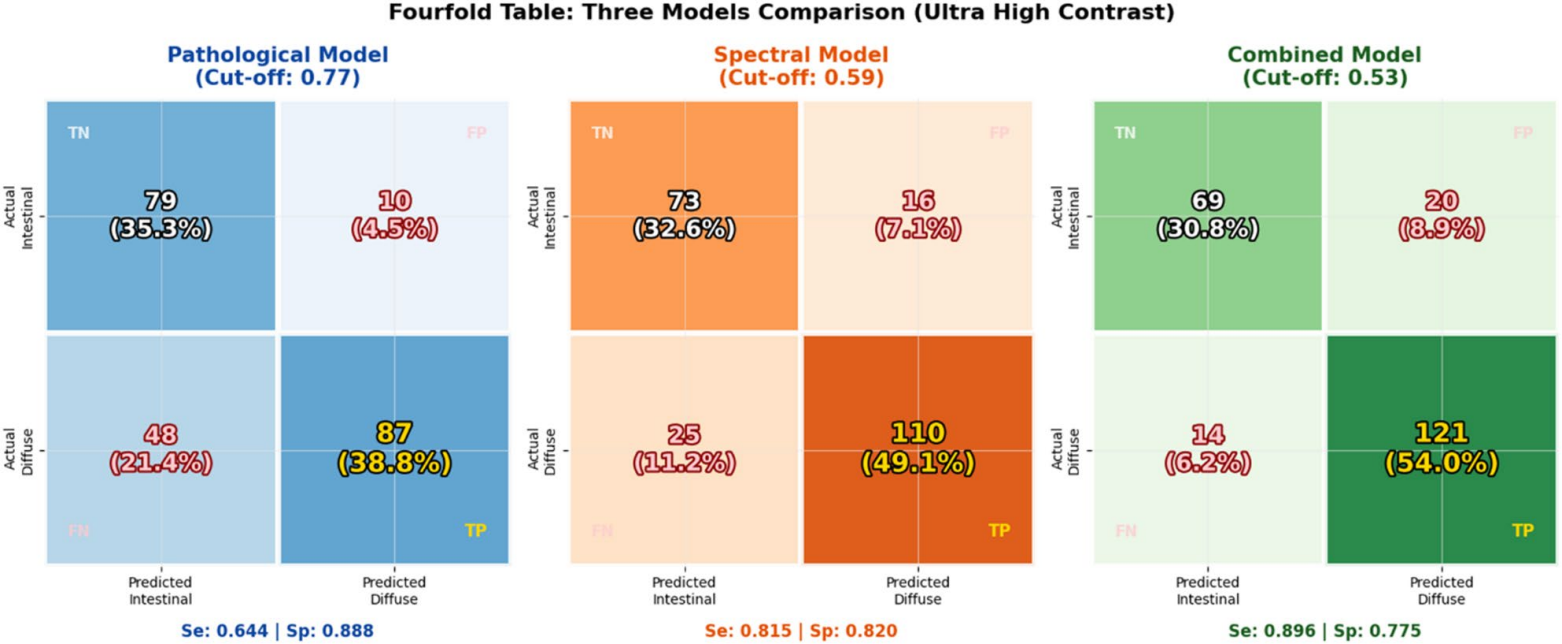
Comparison of confusion matrices for predicting gastric cancer Lauren classification (intestinal vs. diffuse type) among pathological model, spectral CT model, and combined model

**Fig. 2:**
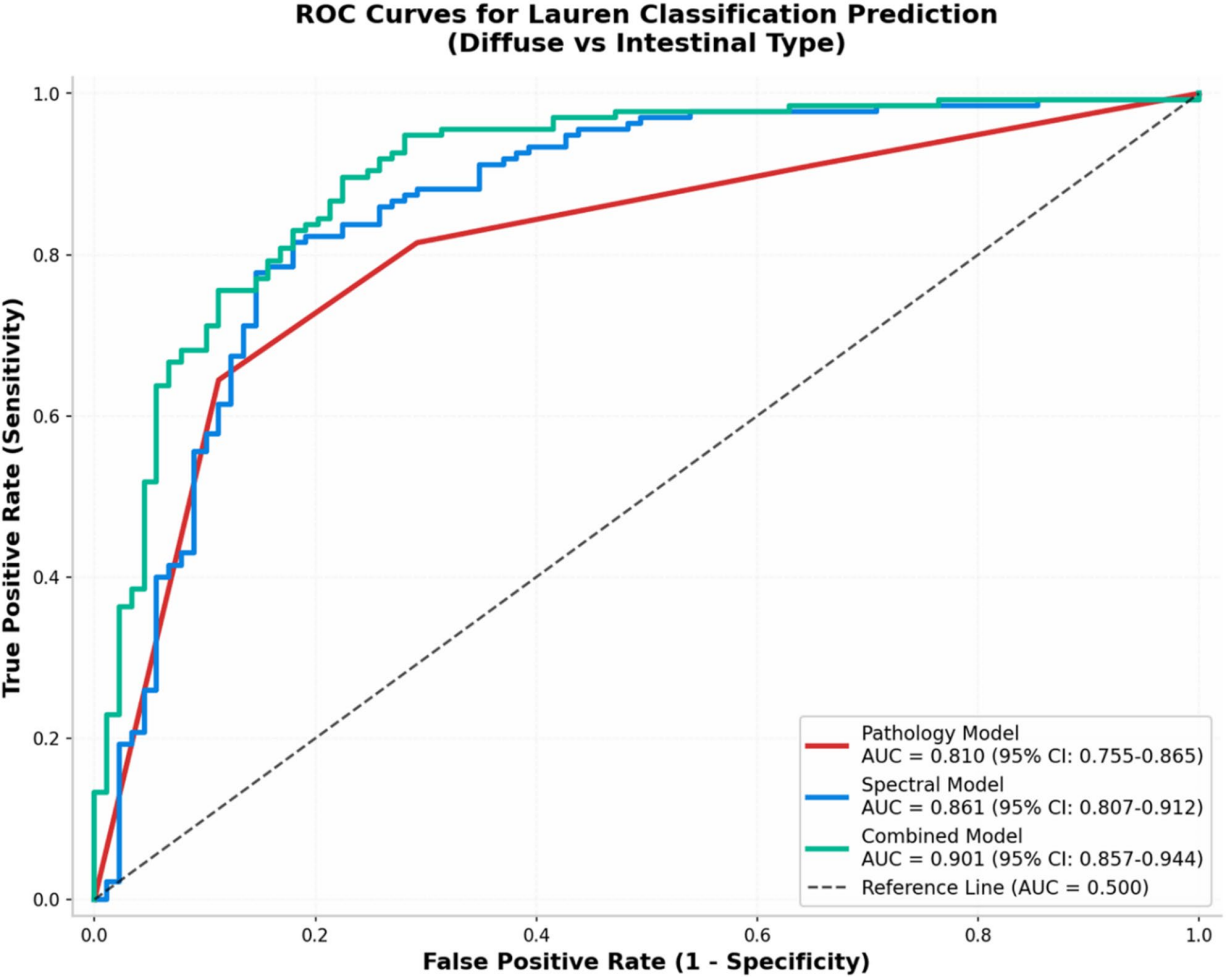
Receiver operating characteristic (ROC) curves of pathological model, spectral CT model, and combined model for predicting gastric cancer Lauren classification

**Fig. 3:**
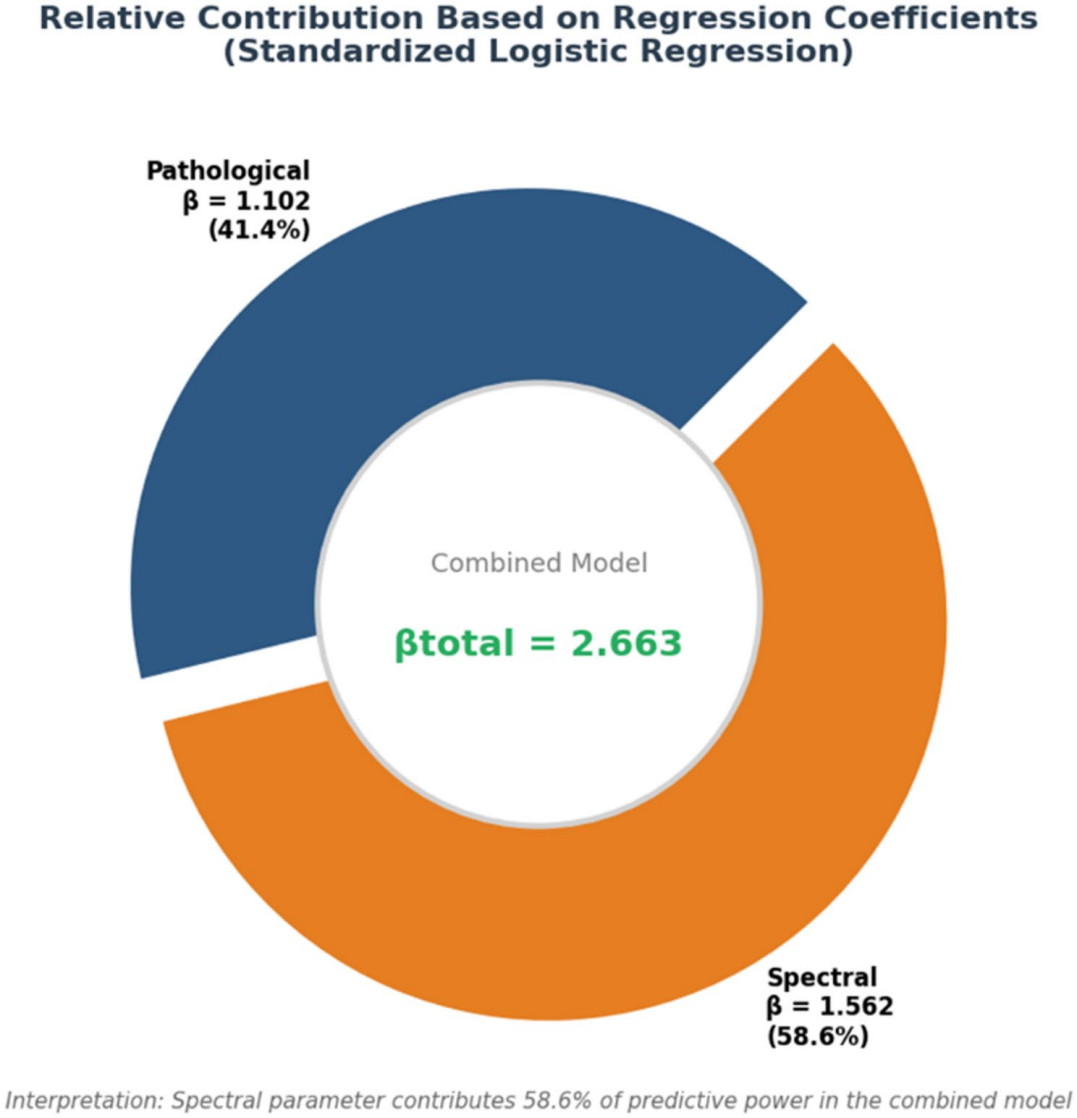
Relative contribution of pathological parameters and spectral CT parameters to the combined model for predicting gastric cancer Lauren classification

**Fig. 4:**
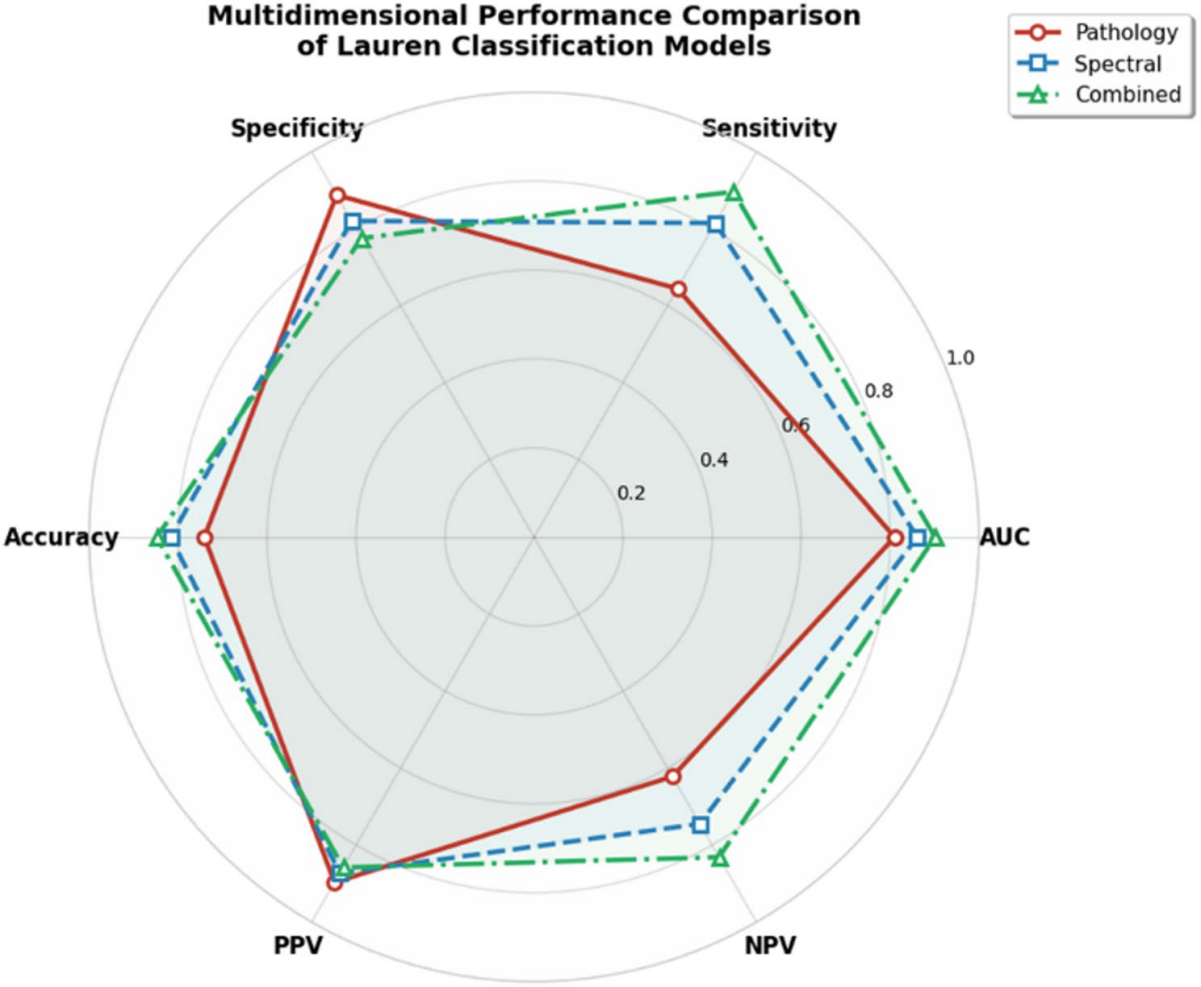
Radar chart comparing the multidimensional diagnostic performance of pathological model, spectral CT model, and combined model for predicting gastric cancer Lauren classification

**Fig. 5:**
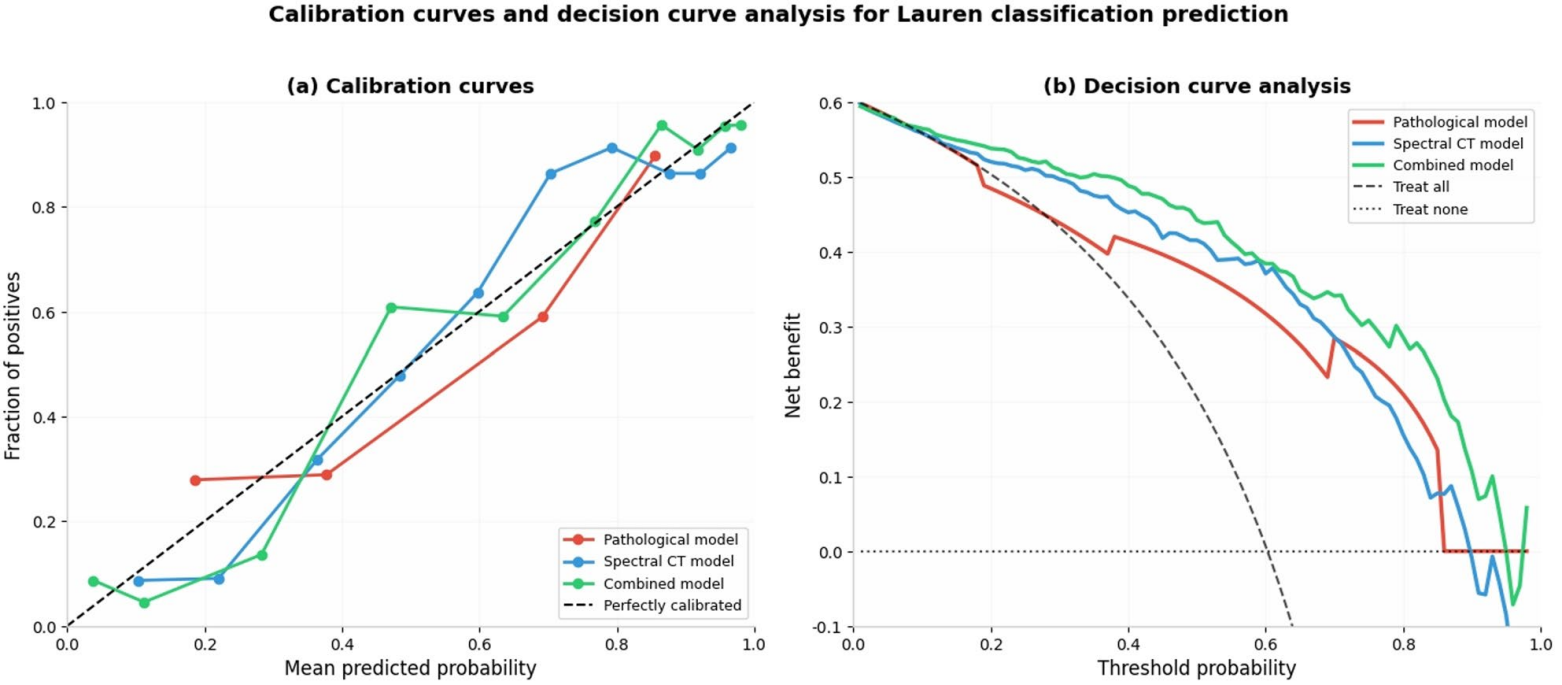
Calibration curves (**a**) and decision curve analysis (**b**) for Lauren classification prediction

Based on the multivariate analyses, the clinicopathological model included poor differentiation and Borrmann type III/IV (Table 2), the spectral CT model included PVP-CT₇₀ₖₑ_v_, PVP-IC, and PVP-NIC (Table 4), and the combined model integrated all five independent predictors identified above. The diagnostic performance of these three models was compared as follows. The combined model achieved an AUC of 0.901 (95% CI: 0.859–0.944), compared to 0.810 (95% CI: 0.752–0.869) for the pathological model and 0.861 (95% CI: 0.808–0.915) for the spectral CT model. The difference in AUC between the combined and pathological models was 0.091 (*P* < 0.001), and between the combined and spectral CT models was 0.040 (*P* = 0.024) (Tables 8 and 9). At a cut-off value of 0.53, the combined model demonstrated sensitivity of 89.6%, specificity of 77.5%, accuracy of 84.8%, positive predictive value of 85.8%, negative predictive value of 83.1%, and a Youden index of 0.671 (Table 8). Classification outcomes for the combined model included 121 true positives, 20 false positives, 69 true negatives, and 14 false negatives (Fig. 1). “ROC curves indicated AUC values of 0.810 for the pathological model, 0.861 for the spectral model, and 0.901 for the combined model (Fig. 2). Standardized regression coefficients were β = 1.562 (58.6%) for spectral CT parameters and β = 1.102 (41.4%) for pathological parameters (Fig. 3).” The radar chart for the combined model indicated sensitivity of 0.896, specificity of 0.775, accuracy of 0.848, AUC of 0.901, positive predictive value of 0.858, and negative predictive value of 0.831 (Fig. 4)0.8, and NPV 0.831 (Fig. 4). Calibration curve analysis demonstrated satisfactory agreement between predicted probabilities and observed outcomes for the combined model (Fig. 5a), and decision curve analysis indicated that the combined model yielded a superior net benefit compared with both the “treat-all” and “treat-none” strategies across threshold probabilities ranging from 10% to 80% (Fig. 5b).Internal validation using 1000-bootstrap resampling confirmed the stability of the combined model, yielding an optimism-corrected AUC of 0.901 (95% CI: 0.857–0.943) for Lauren classification(Table 10).

**Table 8:** Diagnostic performance of different parameters for predicting Lauren-type in gastric cancer

Parameter	Type	AUC(95%CI)	Youden J	Cut-off	Se (%)	Sp (%)	Acc (%)	PPV(%)	NPV(%)
Pathological Parameter	Binary	0.810(0.752,0.869)	0.532	0.77	64.4	88.8	74.1	89.7	62.2
Spectral Parameter	Continuous	0.861(0.808,0.915)	0.635	0.59	81.5	82.0	81.7	87.3	74.5
Combined Parameter	Combined	0.901(0.859,0.944)	0.671	0.53	89.6	77.5	84.8	85.8	83.1

Legend: Abbreviations: AUC, area under the curve; CI, confidence interval; Youden index (Youden J); PPV, positive predictive value; NPV, negative predictive value. The clinicopathological model included tumor differentiation and Borrmann type. The spectral CT model included portal-venous phase CT₇₀ₖₑ_v_, iodine concentration (IC), and normalized iodine concentration (NIC). The combined model integrated all five independent predictors retained in the respective multivariate analyses

**Table 9:** Pairwise comparison of AUCs between Combined Parameter and other parameters for predicting Lauren-type in gastric cancer

Comparison	AUC difference	Youden J difference	z	*p* value	95%CI
Pathological vs. Combined Parameter	0.091	0.139	-3.691	<0.001^*^	0.043 ~ 0.138
Spectral vs. Combined Parameter	0.040	0.036	-2.265	0.024^*^	0.007 ~ 0.076

Legend: Abbreviations: AUC, area under the curve; CI, confidence interval

**Table 10:** Results of 1000-bootstrap internal validation for the combined prediction models

Model	Original AUC	Optimism	Optimism-corrected AUC	Bootstrap 95% CI
Combined model for Lauren classification	0.901	0.000	0.901	0.857–0.943
Combined model for DFS prediction	0.896	0.001	0.895	0.850–0.939

### Diagnostic performance for disease-free survival

**Fig. 6:**
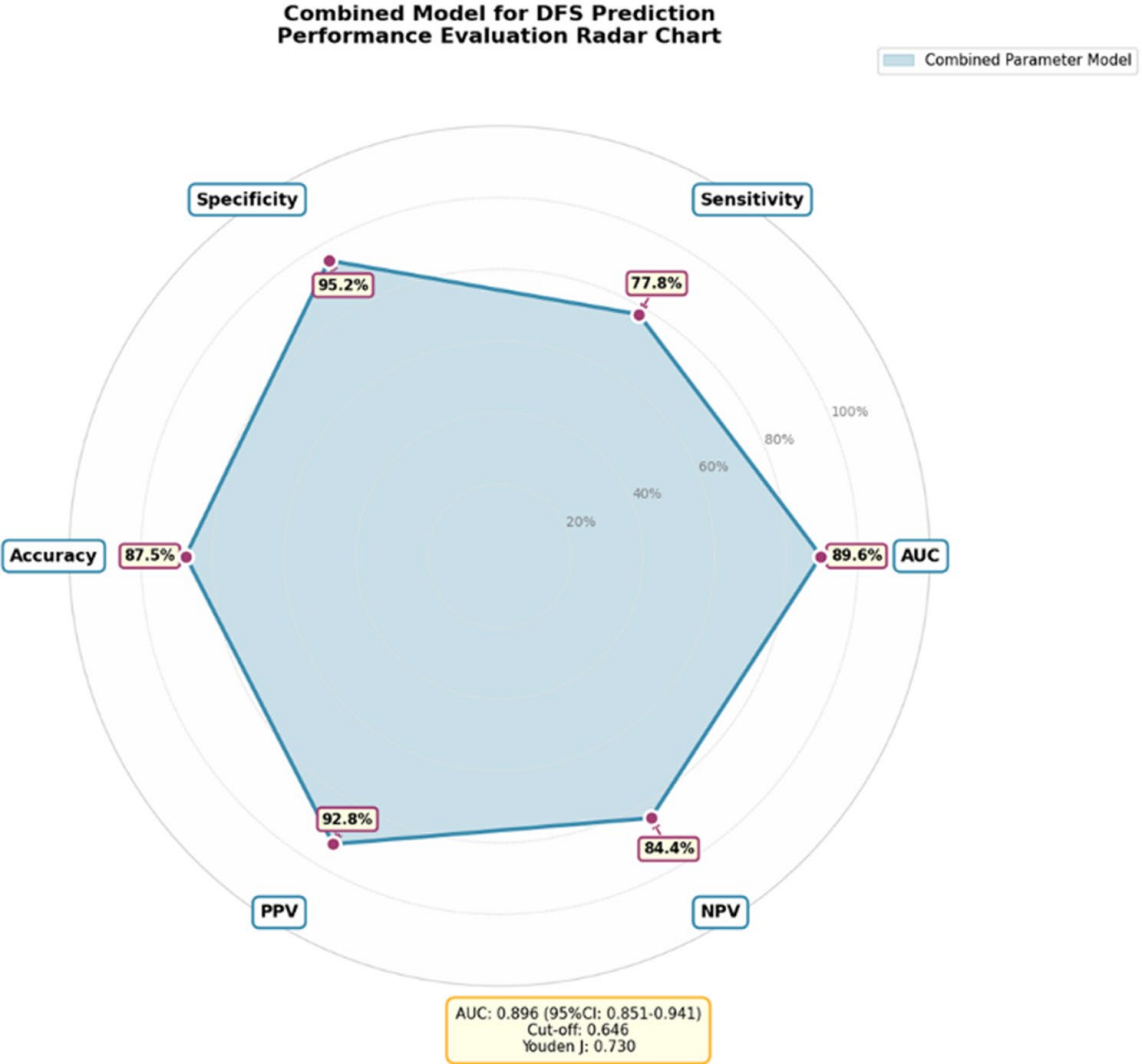
Radar chart showing the diagnostic performance of combined model for predicting disease-free survival (DFS) in gastric cancer patients

**Fig. 7:**
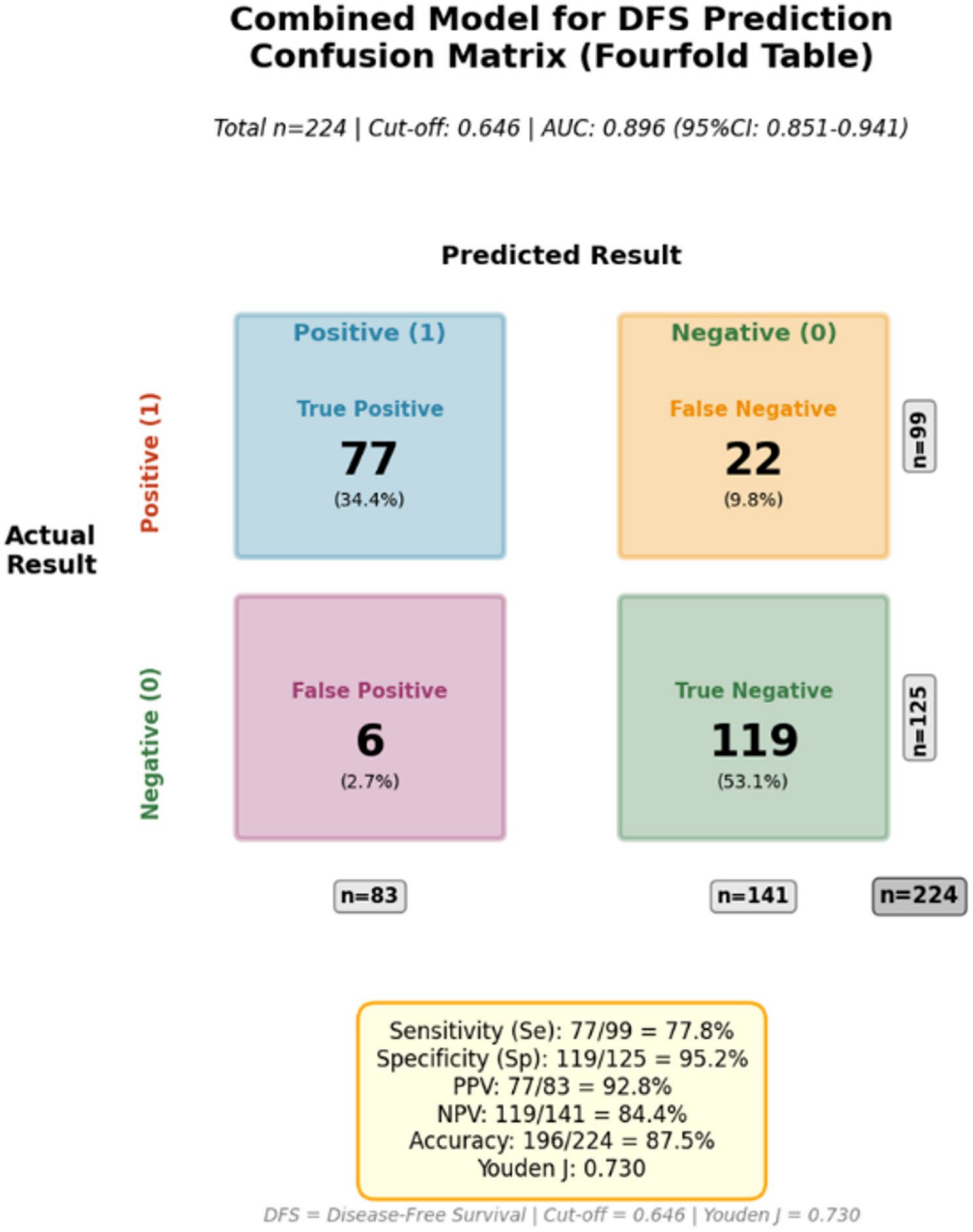
Confusion matrix of combined model for predicting disease-free survival (DFS) in gastric cancer patients

**Fig. 8:**
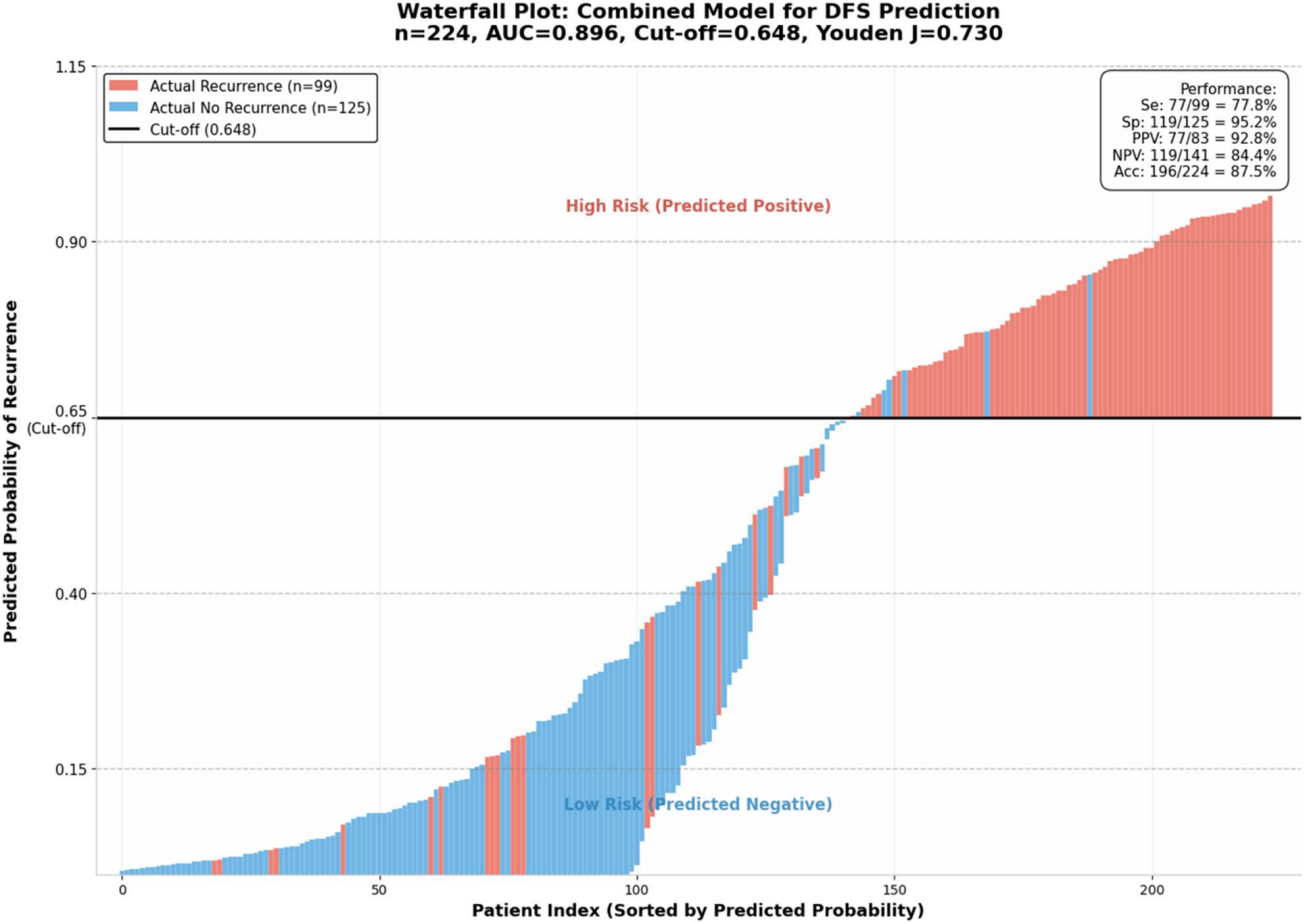
Waterfall plot of combined model for predicting disease-free survival (DFS) in gastric cancer patients

For predicting disease-free survival (DFS), the combined model achieved an AUC of 0.896 (95% CI: 0.851–0.941) at a cut-off value of 0.646 (Figs. 6 and 7). The model demonstrated sensitivity of 77.8%, specificity of 95.2%, accuracy of 87.5%, positive predictive value of 92.8%, negative predictive value of 84.4%, and a Youden index of 0.730 (Fig. 7). The waterfall plot indicated that 99 patients had a predicted probability greater than 0.646, of whom 77 experienced recurrence; 125 patients had a predicted probability of 0.646 or less, of whom 119 did not experience recurrence (Fig. 8).

**Fig. 9:**
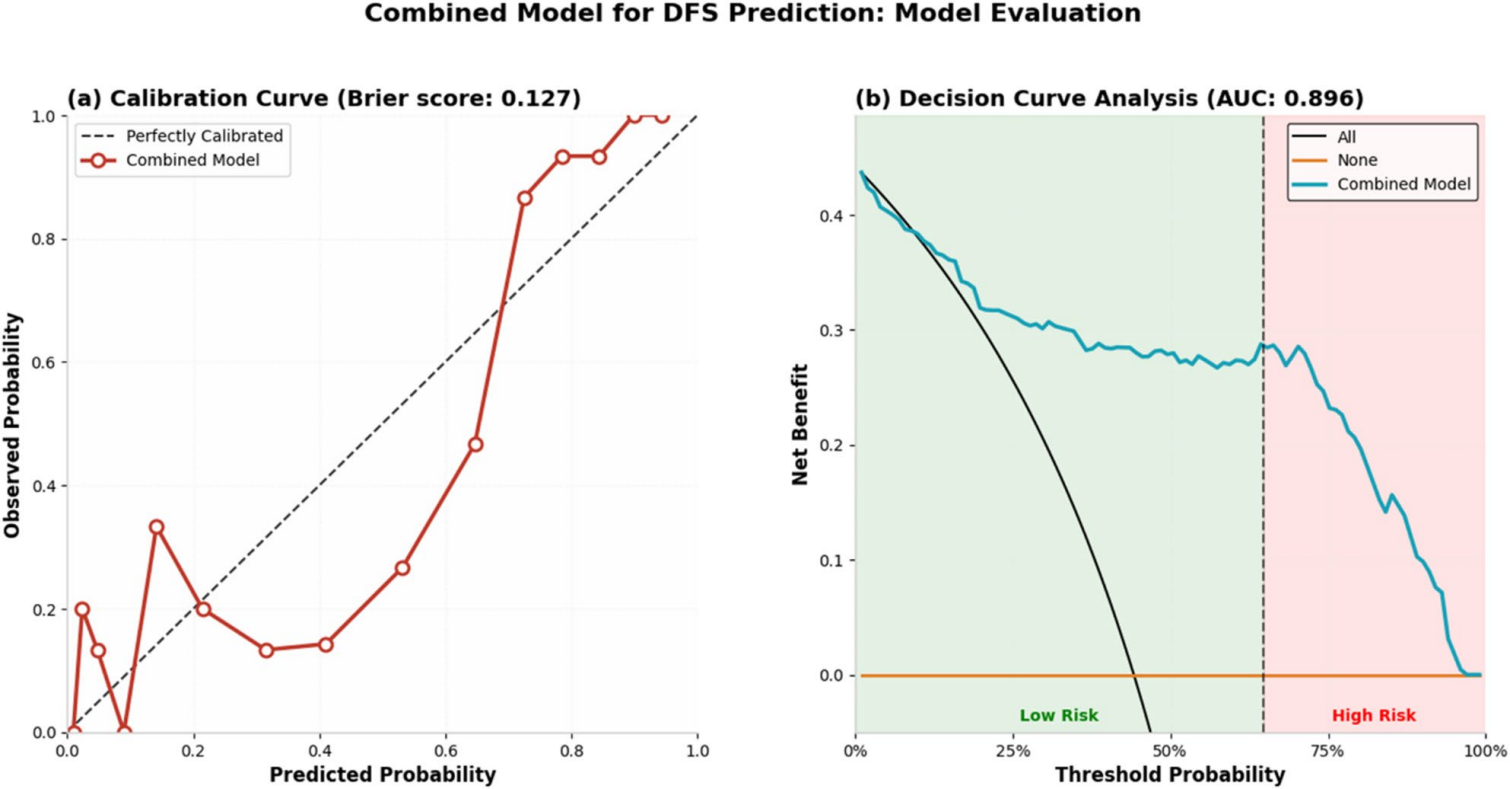
Calibration curve (**a**) and decision curve analysis (**b**) of combined model for predicting disease-free survival (DFS) in gastric cancer patients

**Fig. 10:**
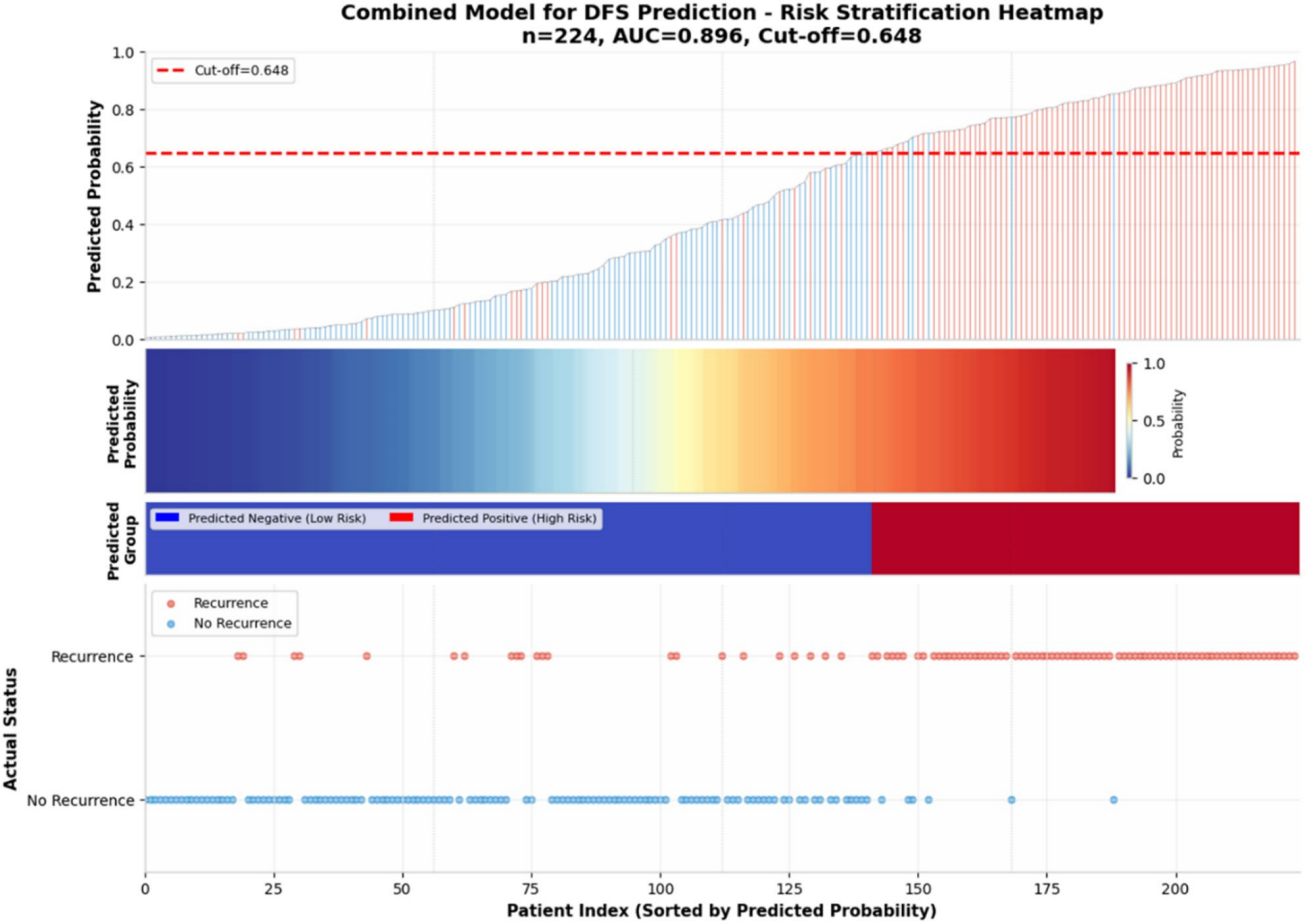
Risk stratification heatmap of combined model for predicting disease-free survival (DFS) in gastric cancer patients

The calibration curve yielded a Brier score of 0.127 (Fig. 9a). Decision curve analysis demonstrated that the net benefit curve for the combined model was superior to both the “treat-all” and “treat-none” strategies across threshold probabilities ranging from 10% to 80% (Fig. 9b). The risk stratification heatmap indicated 141 patients in the high-risk group and 83 in the low-risk group based on the 0.646 cut-off; among these, 77 actual recurrence cases were in the high-risk group, and 119 actual no-recurrence cases were in the low-risk group (Fig. 10).

**Fig. 11:**
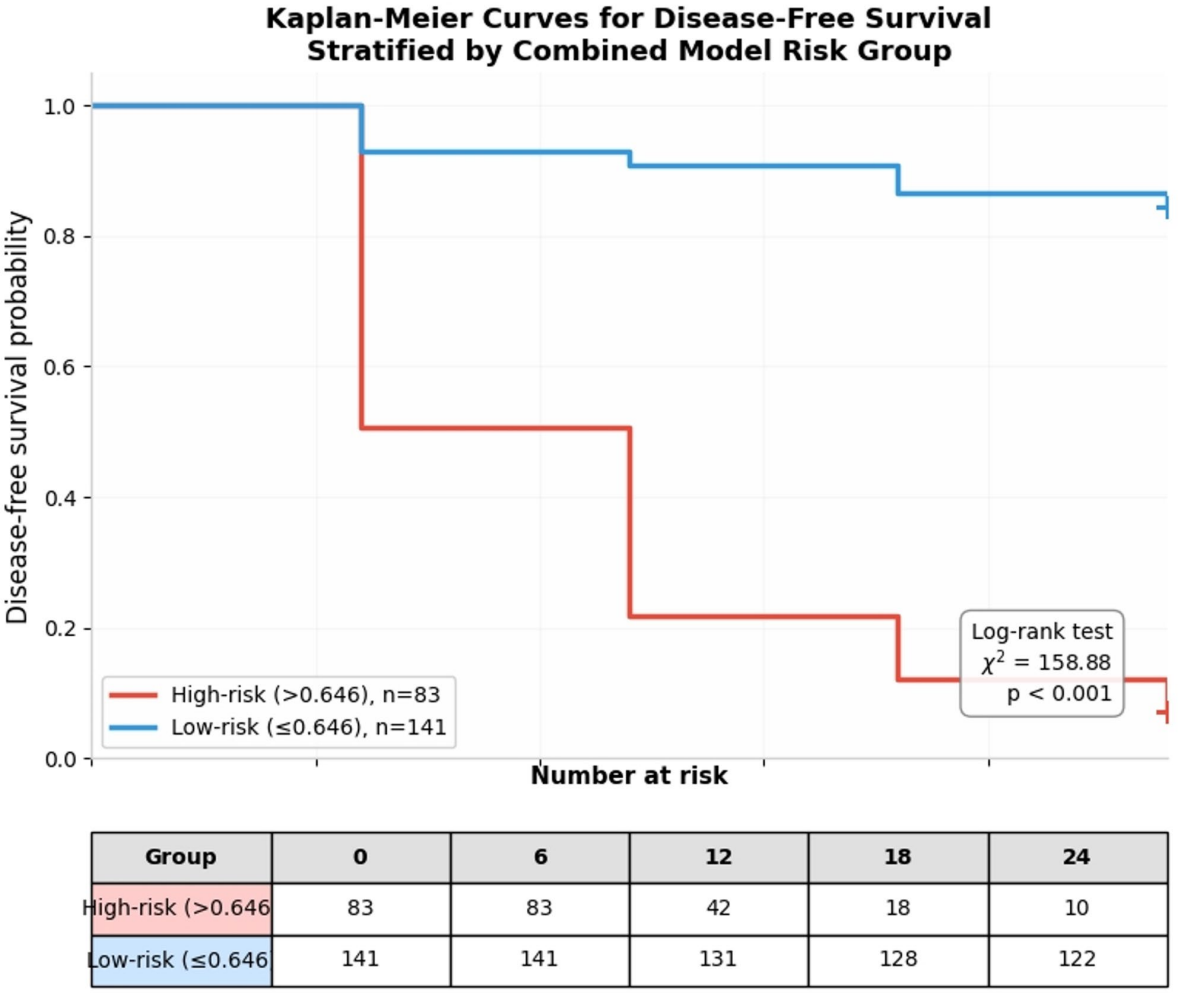
Kaplan-Meier curves for disease-free survival stratified by combined model risk group

Risk stratification and survival analysis. Based on the optimal cut-off value of 0.646, patients were stratified into high-risk (*n* = 83) and low-risk (*n* = 141) groups. Kaplan-Meier analysis demonstrated a significant separation in disease-free survival between the two groups (log-rank χ² = 158.88, *P* < 0.001), with 2-year DFS rates of 7.2% and 84.4%, respectively (Fig. 11). Univariate Cox regression confirmed that patients in the high-risk group exhibited a significantly increased risk of recurrence compared with the low-risk group (HR = 10.97, 95% CI: 6.68–17.99, *P* < 0.001). Similarly, bootstrap internal validation demonstrated an optimism-corrected AUC of 0.895 (95% CI: 0.850–0.939) for DFS prediction, indicating minimal overfitting(Table 10).

## Discussion

### Summary of principal findings

An integrated preoperative prediction model was developed and validated by combining preoperatively available clinicopathological characteristics with portal-venous phase spectral CT parameters for preoperative Lauren classification and disease-free survival (DFS) prediction in gastric cancer patients undergoing chemotherapy. The combined model demonstrated superior performance compared to standalone pathological and spectral CT models, achieving an AUC of 0.901 (95% CI: 0.859–0.944) for distinguishing intestinal from diffuse-type histology and 0.896 (95% CI: 0.851–0.941) for predicting two-year DFS. Multivariate analysis identified poor differentiation (OR = 9.80) and Borrmann type III/IV (OR = 2.65) as principal pathological predictors of diffuse-type histology. Portal-venous phase spectral parameters, specifically CT₇₀ₖₑ_v_, iodine concentration (IC), and normalized iodine concentration (NIC), were independent imaging biomarkers. Spectral CT parameters accounted for 58.6% of the model’s predictive power, while pathological factors contributed 41.4%. These results underscore the significant added value of quantitative imaging biomarkers in characterizing tumor microenvironmental heterogeneity beyond conventional histopathological assessment.

### Lauren classification and the importance of non-invasive assessment

The Lauren classification system is fundamental for gastric cancer prognostication and therapeutic stratification because of the distinct biological behaviors exhibited by intestinal-type tumors, which are characterized by cohesive growth, glandular formation, and a favorable prognosis, in contrast to diffuse-type tumors, which are marked by loss of cell adhesion, signet-ring cells, and aggressive metastasis [23, 24]. These subtypes exhibit differential responses to systemic therapy, as diffuse-type tumors frequently exhibit primary resistance to conventional fluoropyrimidine-platinum chemotherapy and demonstrate early peritoneal dissemination [4, 6]. Current diagnostic reliance on endoscopic biopsy or surgical specimens has significant limitations, including sampling heterogeneity and spatial variation that may misrepresent the entire tumor landscape, particularly in mixed or heterogeneous tumors [8, 9].

### Biological basis of spectral CT signatures

The superiority of portal-venous phase parameters compared to arterial-phase parameters reflects fundamental aspects of tumor biology. The portal-venous phase assesses contrast agent distribution within the tumor microvasculature and interstitial space, thereby capturing desmoplastic stromal reactions and altered perfusion characteristics typical of diffuse-type gastric cancer [10, 12]. Diffuse-type tumors exhibited significantly higher CT₇₀ₖₑ_v_, iodine concentration (IC), and normalized iodine concentration (NIC) compared to intestinal-type lesions (Table 3), consistent with their distinct pathophysiological profiles and previous reports on iodine-based tissue characterization [11, 25]. Intestinal-type tumors typically display organized glandular structures with extensive vascular networks facilitating homogeneous iodine uptake, whereas diffuse-type tumors demonstrate enhanced contrast retention attributable to dense fibrous stroma, heterogeneous vascularity, and increased vascular permeability. These imaging features correlate with established macroscopic classifications, as Borrmann types III and IV predominantly correspond to diffuse-type histology with infiltrative growth patterns [16].

### Structure and diagnostic efficacy of the integrative model

The incremental benefit of integrating spectral CT with clinicopathological features is evidenced by pairwise AUC comparisons (Table 9). The combined model demonstrated a significant AUC increase compared to pathological or imaging models alone, achieving optimal sensitivity and balanced specificity [10, 26]. This improvement is attributable to the complementary contributions of the included variables: whereas Borrmann type and differentiation grade represent macroscopic growth patterns and cellular organization, spectral CT parameters evaluate tumor vascularity and iodine metabolism at the microenvironmental level [15, 27]. The high negative predictive value of the combined model for Lauren classification suggests particular utility in excluding diffuse-type histology, potentially reducing unnecessarily aggressive treatment protocols for patients with intestinal-type tumors, similar to the risk stratification approach used for occult peritoneal metastasis identification [28].

### Comparison with existing prediction models

These findings should be considered in light of recent advances in preoperative prognostication for gastric cancer. Li et al. [10] used CT scans to classify tumors by Lauren type and achieved strong performance; however, their approach relied solely on complex imaging features and did not include basic clinical information, which may limit practical application. Cheng et al. [13] also used CT scans and deep learning to classify tumors and analyze the tumor microenvironment. Although their method demonstrated high accuracy and automated feature analysis, it required significant computational resources and lacked transparency. Cao et al. [26] provided additional evidence through a multicenter study employing automated CT scanning and image analysis, emphasizing the need for standardized feature measurement. Dong et al. [28] combined CT images with clinical data to develop a chart for detecting occult peritoneal metastasis, illustrating the value of integrated data approaches. In contrast, the present model offers several distinct advantages. It achieves high accuracy in both tumor classification and disease-free survival prediction (AUCs: 0.901 and 0.896) by using straightforward CT features that are easily interpretable and applicable, rather than complex imaging characteristics. Additionally, it simultaneously predicts two critical outcomes: tumor type and prognosis, whereas previous tools typically addressed only one. The model also demonstrates robust consistency with minimal overfitting, as confirmed by repeated validation. Together, these attributes make the model practical and cost-effective for preoperative risk assessment, especially in settings without access to advanced imaging analysis or deep learning infrastructure.

### Ensuring methodological rigor and reproducibility

We implemented several methodological safeguards to strengthen the robustness of our study. Collinearity diagnostics revealed that CT₄₀ₖₑ_v_ exhibited severe multicollinearity (variance inflation factor [VIF] = 12.369) with other spectral parameters, consistent with statistical standards in radiomics research requiring VIF thresholds below 5 for model stability [29, 30]. After excluding CT₄₀ₖₑ_v_, all remaining VIF values decreased to below 5, which stabilized the regression coefficients and reduced the risk of overfitting. Furthermore, the spectral CT parameter measurements showed excellent inter-observer reproducibility (ICC range: 0.871–0.935), supporting the clinical applicability of these biomarkers because they can be reliably quantified by radiologists with varying levels of experience, as demonstrated in texture analysis studies requiring ICC thresholds above 0.75 for feature selection [14].

### Clinical implications of predicting disease-free survival and risk stratification

Beyond histological classification, the combined model exhibited strong predictive performance for disease-free survival, with high specificity and a favorable positive predictive value. Decision curve analysis indicated a superior net benefit across threshold probabilities ranging from 10% to 80% compared to treat-all or treat-none strategies, supporting its clinical utility in risk-stratified surveillance protocols [31, 32]. The model’s capacity to identify high-risk patients who may benefit from intensified adjuvant chemotherapy or closer postoperative monitoring aligns with prognostic stratification strategies used in surgical oncology for treatment allocation [33]. In contrast, low-risk patients may be spared unnecessary treatment-related toxicities through conservative surveillance protocols informed by accurate preoperative risk assessment.

### Broader implications for gastrointestinal oncology

The multimodal strategy employed in this study aligns with recent advancements in oncological imaging for gastrointestinal malignancies. Integrated methodologies that combine quantitative imaging biomarkers with clinicopathological variables are increasingly implemented for preoperative risk stratification in colorectal cancer, hepatocellular carcinoma, and pancreatic ductal adenocarcinoma. For instance, Lisson et al. [29] demonstrated that machine learning-based radiomics can effectively distinguish T2 from T3 bladder cancer, illustrating the cross-tumor applicability of quantitative imaging techniques when rigorous validation standards are applied. In gastric cancer, integrating spectral CT-derived iodine quantification with clinical variables underscores that tumor vascularity and microenvironmental heterogeneity, as measured by contrast-enhanced imaging, are translatable biological features across multiple malignancies. The conceptual framework that incorporates macroscopic growth patterns (Borrmann type), cellular differentiation status, and microvascular iodine metabolism may also be relevant to other gastrointestinal tumors where preoperative histological prediction and prognosis estimation remain challenging, such as colorectal adenocarcinoma and hepatocellular carcinoma. Additional cross-tumor validation studies are required to assess whether the spectral CT parameters identified in the gastric cancer cohort demonstrate similar discriminative power in other gastrointestinal malignancies, potentially establishing a generalized paradigm for multimodal preoperative stratification.

### Limitations and future directions

Several limitations warrant consideration. This retrospective single-center study requires external validation given its moderate sample size. Heterogeneity in chemotherapy regimens was not addressed, and Borrmann classification may exhibit inter-operator variability. We further acknowledge that while spectral CT is non-invasive, tumor differentiation and Borrmann type require histopathological assessment; thus, the model is more accurately characterized as an integrated preoperative prediction tool leveraging routinely available preoperative data, rather than a strictly non-invasive one. However, the spectral CT component contributed 58.6% of the predictive power, representing the genuinely non-invasive incremental value. Future research should prioritize prospective validation in randomized controlled trials, integrate radiomics and deep learning to achieve fully non-invasive histological prediction, and correlate imaging signatures with molecular biomarkers (e.g., PD-L1, HER2, EBV) to develop multi-omics models for precision oncology.

## Conclusion

This study establishes a robust and non-invasive framework for preoperative Lauren classification and disease-free survival (DFS) prediction in gastric cancer by integrating clinicopathological features with portal-venous phase spectral CT parameters. The superior performance of the integrated model compared to individual diagnostic modalities highlights the significance of multimodal assessment in capturing the biological complexity of gastric cancer. As healthcare systems increasingly prioritize precision medicine, quantitative imaging biomarkers are likely to become valuable tools for treatment planning, prognostication, and dynamic monitoring, ultimately contributing to improved patient outcomes through personalized therapeutic strategies.

## Data Availability

The datasets generated and/or analyzed during the current study are not publicly available due to patient privacy restrictions and institutional policy, but are available from the corresponding author on reasonable request.

## References

[1] Sung H, Ferlay J, Siegel RL, Laversanne M, Soerjomataram I, Jemal A, et al. Global Cancer Statistics 2020: GLOBOCAN Estimates of Incidence and Mortality Worldwide for 36 Cancers in 185 Countries. CA Cancer J Clin. 2021;71(3):209–49. 33538338 10.3322/caac.21660

[2] Xiao M, Qian LL, Zhao Q, Ma J, Li Q, Liu Q, et al. Changes in thyroid hormone levels indicate immunotherapy efficacy in gastric cancer. Oncol Lett. 2025;30(1):364. 40469917 10.3892/ol.2025.15110 PMC12134976

[3] Zheng R, Zhang S, Zeng H, Wang S, Sun K, Chen R, et al. Cancer incidence and mortality in China, 2016. J Natl Cancer Cent. 2022;2(1):1–9. 39035212 10.1016/j.jncc.2022.02.002 PMC11256658

[4] Pernot S, Voron T, Perkins G, Lagorce-Pages C, Berger A, Taieb J. Signet-ring cell carcinoma of the stomach: Impact on prognosis and specific therapeutic challenge. World J Gastroenterol. 2015;21(40):11428–38. 26523107 10.3748/wjg.v21.i40.11428 PMC4616218

[5] Kawazoe A, Kuwata T, Kuboki Y, Shitara K, Nagatsuma AK, Aizawa M, et al. Clinicopathological features of programmed death ligand 1 expression with tumor-infiltrating lymphocyte, mismatch repair, and Epstein-Barr virus status in a large cohort of gastric cancer patients. Gastric Cancer. 2017;20(3):407–15. 27629881 10.1007/s10120-016-0631-3

[6] Ebrahim NAA, Othman MO, Tahoun NS, Salama RA, Arafat A, Amin NH. Deciphering prognostic markers in gastric signet ring cell carcinoma: Human epidermal growth factor receptor 2 and other key factors. World J Clin Oncol. 2025;16(8):107987. 40901316 10.5306/wjco.v16.i8.107987 PMC12400182

[7] Qiu MZ, Shi SM, Chen M, Wang J, Wu QN, Sheng H, et al. Comparison of HER2 and Lauren Classification between Biopsy and Surgical Resection Samples, Primary and Metastatic Samples of Gastric Cancer. J Cancer. 2017;8(17):3531–7. 29151938 10.7150/jca.19984 PMC5687168

[8] Rosanu NM, Gervaso L, Lobrano R, Vanoli A, Cella CA, Fusco N et al. Diagnostic and therapeutic challenges related to her2 heterogeneity in gastric cancer: bridging molecular pathology and clinical decision-making. Int J Mol Sci. 2026;27(3). 10.3390/ijms27031542 PMC12898168 41683961

[9] Soh JS, Lim H, Kang HS, Kim JH, Kim KC. Does the discrepancy in histologic differentiation between a forceps biopsy and an endoscopic specimen necessitate additional surgery in early gastric cancer? World J Gastrointest Oncol. 2017;9(8):319–26. 28868112 10.4251/wjgo.v9.i8.319 PMC5561043

[10] Li M, Qin H, Yu X, Sun J, Xu X, You Y, et al. Preoperative prediction of Lauren classification in gastric cancer: a radiomics model based on dual-energy CT iodine map. Insights Imaging. 2023;14(1):125. 37454355 10.1186/s13244-023-01477-8 PMC10350444

[11] Li R, Li J, Wang X, Liang P, Gao J. Detection of gastric cancer and its histological type based on iodine concentration in spectral CT. Cancer Imaging. 2018;18(1):42. 30413174 10.1186/s40644-018-0176-2 PMC6230291

[12] Liang P, Ren XC, Gao JB, Chen KS, Xu X. Iodine Concentration in Spectral CT: Assessment of Prognostic Determinants in Patients With Gastric Adenocarcinoma. AJR Am J Roentgenol. 2017;209(5):1033–8. 28871809 10.2214/AJR.16.16895

[13] Cheng M, Guo Y, Zhao H, Zhang H, Liang P, Gao J. CT-based deep learning radiomics analysis for preoperative Lauren classification in gastric cancer and explore the tumor microenvironment. Eur J Radiol Open. 2025;15:100667. 40607047 10.1016/j.ejro.2025.100667 PMC12221457

[14] Liu S, Shi H, Ji C, Guan W, Chen L, Sun Y, et al. CT textural analysis of gastric cancer: correlations with immunohistochemical biomarkers. Sci Rep. 2018;8(1):11844. 30087428 10.1038/s41598-018-30352-6 PMC6081398

[15] Wang XX, Ding Y, Wang SW, Dong D, Li HL, Chen J, et al. Intratumoral and peritumoral radiomics analysis for preoperative Lauren classification in gastric cancer. Cancer Imaging. 2020;20(1):83. 33228815 10.1186/s40644-020-00358-3 PMC7684959

[16] Del Díaz C, Ortega Medina L, Estrada Muñoz L, Molina Roldán E, Cerón Nieto M, et al. García Gómez de Las Heras S,. Are Borrmann’s Types of Advanced Gastric Cancer Distinct Clinicopathological and Molecular Entities? A Western Study. Cancers (Basel). 2021;13(12). 10.3390/cancers13123081 PMC8234739 34205546

[17] Ng D, Cyr D, Khan S, Dossa F, Swallow C, Kazazian K. Molecular mechanisms of metastatic peritoneal dissemination in gastric adenocarcinoma. Cancer Metastasis Rev. 2025;44(2):50. 40317360 10.1007/s10555-025-10265-3 PMC12049340

[18] Yasumoto M, Sakamoto E, Ogasawara S, Isobe T, Kizaki J, Sumi A, et al. Muscle RAS oncogene homolog (MRAS) recurrent mutation in Borrmann type IV gastric cancer. Cancer Med. 2017;6(1):235–44. 27891760 10.1002/cam4.959 PMC5269692

[19] Siriwardana A, Smyth B, Jardine M. Waiver of informed consent in clinical research: a summary of contemporary guidelines and a resource for researchers. BMJ Open. 2025;15(3):e091896. 40107704 10.1136/bmjopen-2024-091896 PMC11927427

[20] World Medical Association Declaration of Helsinki. Ethical Principles for Medical Research Involving Human Participants. JAMA. 2025;333(1):71–4. 39425955 10.1001/jama.2024.21972

[21] Bossuyt PM, Reitsma JB, Bruns DE, Gatsonis CA, Glasziou PP, Irwig L, et al. STARD 2015: an updated list of essential items for reporting diagnostic accuracy studies. BMJ. 2015;351:h5527. 26511519 10.1136/bmj.h5527 PMC4623764

[22] Collins GS, Reitsma JB, Altman DG, Moons KG. Transparent reporting of a multivariable prediction model for individual prognosis or diagnosis (TRIPOD): the TRIPOD statement. BMJ. 2015;350:g7594. 25569120 10.1136/bmj.g7594

[23] Jiménez Fonseca P, Carmona-Bayonas A, Hernández R, Custodio A, Cano JM, Lacalle A, et al. Lauren subtypes of advanced gastric cancer influence survival and response to chemotherapy: real-world data from the AGAMENON National Cancer Registry. Br J Cancer. 2017;117(6):775–82. 28765618 10.1038/bjc.2017.245 PMC5589993

[24] Ooki A, Yamaguchi K. The dawn of precision medicine in diffuse-type gastric cancer. Ther Adv Med Oncol. 2022;14:17588359221083049. 35281349 10.1177/17588359221083049 PMC8908406

[25] Chen XH, Ren K, Liang P, Chai YR, Chen KS, Gao JB. Spectral computed tomography in advanced gastric cancer: Can iodine concentration non-invasively assess angiogenesis? World J Gastroenterol. 2017;23(9):1666–75. 28321168 10.3748/wjg.v23.i9.1666 PMC5340819

[26] Cao B, Hu J, Li H, Liu X, Rong C, Li S, et al. Preoperative prediction of the Lauren classification in gastric cancer using automated nnU-Net and radiomics: a multicenter study. Insights Imaging. 2025;16(1):48. 40000513 10.1186/s13244-025-01923-9 PMC11861772

[27] Chen Z, Zhang G, Liu Y, Zhu K. Radiomics analysis in predicting vascular invasion in gastric cancer based on enhanced CT: a preliminary study. BMC Cancer. 2024;24(1):1020. 39152398 10.1186/s12885-024-12793-7 PMC11330039

[28] Dong D, Tang L, Li ZY, Fang MJ, Gao JB, Shan XH, et al. Development and validation of an individualized nomogram to identify occult peritoneal metastasis in patients with advanced gastric cancer. Ann Oncol. 2019;30(3):431–8. 30689702 10.1093/annonc/mdz001 PMC6442651

[29] Lisson CG, Gallee L, Müller K, Manoj S, Stöckl H, Zengerling F, et al. Machine learning-based radiomics for bladder cancer staging: evaluating the role of imaging timing in differentiating T2 from T3 disease. Front Oncol. 2025;15:1591742. 41079089 10.3389/fonc.2025.1591742 PMC12512170

[30] Wang J, Tang S, Mao Y, Wu J, Xu S, Yue Q, et al. Radiomics analysis of contrast-enhanced CT for staging liver fibrosis: an update for image biomarker. Hepatol Int. 2022;16(3):627–39. 35347597 10.1007/s12072-022-10326-7 PMC9174317

[31] Wu J, Wang J, Chen N, Nie J, Xia L, Li Q, et al. A prognostic nomogram for predicting overall survival in gastric signet ring cell carcinoma patients: a SEER database and Chinese registry analysis. Front Mol Biosci. 2025;12:1704157. 41333052 10.3389/fmolb.2025.1704157 PMC12665595

[32] Yu KX, Li J, Wang HZ, Zhang CY, Ma MD, Xiao L, et al. Prognostic model for the prediction of cancer-specific survival in elderly patients with stage I-III gastric cancer. Am J Transl Res. 2023;15(5):3188–202. 37303666 PMC10251024

[33] Sarela AI, Lefkowitz R, Brennan MF, Karpeh MS. Selection of patients with gastric adenocarcinoma for laparoscopic staging. Am J Surg. 2006;191(1):134–8. 16399124 10.1016/j.amjsurg.2005.10.015

